# Trends in oncologic hybrid imaging

**DOI:** 10.1186/s41824-017-0019-6

**Published:** 2018-01-19

**Authors:** Andreas G. Wibmer, Hedvig Hricak, Gary A. Ulaner, Wolfgang Weber

**Affiliations:** 1 Department of Radiology, Memorial Sloan Kettering Cancer Center, 1275 York Avenue, New York, NY 10065 USA; 2 Molecular Imaging and Therapy Service, Memorial Sloan Kettering Cancer Center, 1275 York Avenue, New York, NY 10065 USA

**Keywords:** Oncologic hybrid molecular imaging, Time-of-flight positron emission tomography computed tomography, One-minute whole-body PET explorer, ^18^F –Fluciclovine, ^11^C–choline, Prostate-specific membrane antigen, ^18^F–Fluorodehydrotestosterone, ^89^Zr-trastuzumab, ^18^F–Fluoroestradiol, ^68^Ga/ ^177^Lu -DOTA-TATE

## Abstract

Hybrid imaging plays a central role in the diagnosis and management of a wide range of malignancies at all stages. In this article, we review the most pertinent historical developments, emerging clinical applications of novel radiotracers and imaging technologies, and potential implications for training and practice. This includes an overview of novel tracers for prostate, breast, and neuroendocrine tumors, assessment of tumor heterogeneity, the concept of image-guided ‘biologically relevant dosing’, and theranostic applications. Recent technological advancements, including time-of-flight PET, PET/MRI, and ‘one-minute whole-body PET’, are also covered. Finally, we discuss how these rapidly evolving applications might affect current training curricula and how imaging-derived big data could be harnessed to the benefit of our patients.

## Background

In biology, a *hybrid* is defined as the offspring of different species, genera or varieties, resulting in an individual that not only combines the qualities of its parents but may exhibit improved qualities or functions, a phenomenon referred to as ‘hybrid vigor’. Similarly, the synergistic combination of anatomical imaging (e.g. computed tomography-CT, magnetic resonance imaging-MRI) and molecular imaging techniques (e.g. positron emission tomography-PET, single photon emission computed tomography-SPECT) can provide a more complete insight into biological processes within their macro-anatomical, often whole-body, environment. While combinations of these techniques have been applied in a broad range of medical disciplines (e.g. oncology, cardiology, neurology, psychiatry, pharmacology), this article focuses on hybrid imaging in oncology, where ^18^F–fluorodeoxyglucose (FDG)-PET/CT accounts for the majority of current applications. With recent and expected approvals of radiotracers, new developments in PET/CT technology, and increased use of PET/MRI, hybrid imaging is expected to cover much more diverse clinical roles in the near future. This review gives an overview of pertinent historical developments, emerging applications, and future trends.

## Historical milestones

**Fig. 1:**
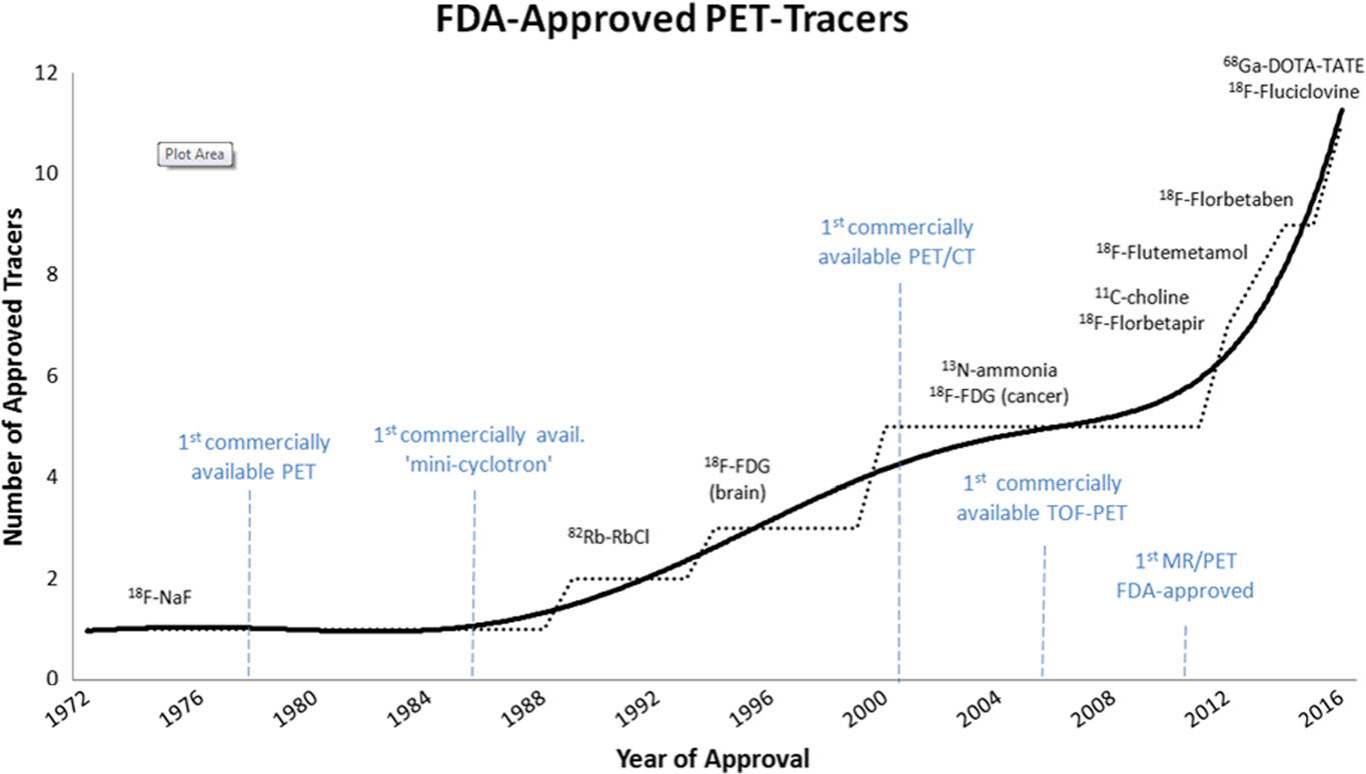
Timeline of Food and Drug Administration (FDA)-approvals of PET tracers and technical milestones, from 1972 to 2016. Abbreviations: NaF: Sodium fluoride, RbCl: Rubidium chloride, FDG: Fluorodeoxyglucose, PET: Positron Emission Tomography, CT: Computed Tomography, MRI: Magnetic Resonance Imaging, TOF: Time-of-Flight

The evolution of hybrid imaging in oncology was driven by both technical and radiopharmaceutical developments (Fig. 1). As early as in 1972, the US Food and Drug Administration (FDA) approved the first positron-emitting radiotracer, ^18^F–sodium fluoride (^18^F–NaF), for bone scintigraphy using planar gamma camera imaging. This was before the first PET scanner became commercially available in 1978. With the commercial availability of self-shielded, low-energy cyclotrons for use in the hospital environment, and combined PET/CT scanners, the potential of PET imaging could be exploited clinically. Over the following nearly three decades, the few newly approved PET tracers were for non-oncologic indications; it was not until the year 2000 that ^18^F–FDG, which had first been administered to a human in 1976 and approved for neurologic studies in 1994, was broadly approved for imaging in patients with cancer. Since then, another seven PET tracers have been approved by the FDA, and additional approvals are expected for myocardial perfusion imaging and imaging of prostate cancer in the near future. This suggests that growth in the number of clinically available molecular imaging probes is accelerating. In Europe, less rigid regulatory provisions on radiopharmaceuticals have allowed for a more rapid transition of novel radiotracers into clinical care during the last years. The latest technical milestones were the market maturity of time-of-flight (TOF) PET systems (2006) and the FDA approval of the first PET/MRI system in 2011. The expanding number of hybrid imaging devices and molecular imaging probes has implications for clinical practice, staff training, image and data analysis, and the way we perform research and clinical studies in oncology.

## Novel tracers and applications

At this time, ^18^F–FDG remains the most thoroughly studied and most widely used tracer in oncologic hybrid imaging due to biological reasons, most importantly the Warburg effect, and technical/practical considerations, e.g., the possibility of on-site synthesis and the tracer’s favorable effective half-life. In the US, ^18^F–FDG has an established role for the initial staging, treatment response assessment, and restaging of a broad range of malignancies. In several countries outside of the US, imaging of prostate cancer with PSMA (prostate-specific membrane antigen) directed agents has become a standard examination for restaging of prostate cancer. While ^18^F–FDG and ^68^Ga- or ^18^F–PSMA ligands will most probably remain the workhorses for oncologic hybrid imaging in the foreseeable future, several other imaging probes are being developed and clinically tested, which are intended to cover clinical scenarios in which ^18^F–FDG and PSMA ligands do not provide sufficient precision in tumor characterization. While a detailed description of all these agents would be widely beyond the scope of this article, the following paragraphs introduce some of them and describe emerging applications of hybrid imaging.

### Prostate cancer

Prostate cancers exhibit variable ^18^F–FDG avidity. While advanced castration-resistant metastatic disease typically shows intense FDG avidity which is similar to that seen in high-grade lymphomas, FDG uptake is typically low in low-grade prostate malignancies and at early clinical states of prostate cancer. Therefore, its clinical value is limited for initial staging and localization of metastases in the setting of biochemical recurrence after prostatectomy or radiation therapy (Liu et al., 2016). Several PET tracers targeting different molecular structures and processes have been developed as alternatives (Wibmer et al., 2016), and two of them, ^11^C–choline and ^18^F–Fluciclovine, have recently been approved by the FDA. In a prospective study directly comparing these two tracers in patients with biochemical relapse after prostatectomy, ^18^F–Fluciclovine was found to detect more recurrent or metastatic lesions than ^11^C–choline PET/CT (Nanni et al., 2016). The performance of both agents was dependent on prostate-specific antigen (PSA) levels, and in patients with a PSA level below 1 ng/mL, the detection rates were 14% for ^11^C–choline and 21% for ^18^F–Fluciclovine, indicating that there is still a need for more sensitive tracers.

Prostate-Specific Membrane Antigen (PSMA), another target for imaging tracers, is a transmembrane protein that is highly overexpressed in prostate cancer compared to normal prostate and non-prostatic tissues (Ristau et al., 2014). Several PSMA-directed radiolabeled agents have been developed, including monoclonal antibodies, antibody-fragments, minibodies, and more recently small molecules. During the last 5 years, small-molecule ligands for PSMA have shown exceptional sensitivity and specificity for detection of recurrent prostate cancer. On the basis of these data, PSMA PET/CT, as noted earlier, is now the de-facto clinical standard for restaging of patients with biochemical recurrence in many countries. For instance, in a retrospective analysis of 1007 patients undergoing PET/CT with a radiolabeled small molecule PSMA-ligand (i.e. ^68^Ga-PSMA-11) for the evaluation of recurrent prostate cancer, the detection rate in patients with PSA levels ≤1 ng/ml was reported to be 57% (Afshar-Oromieh et al., 2017). In another study of 48 patients with biochemical recurrence, ^68^Ga-PSMA PET detected 53 of 68 pathology-proven lymph node metastases, yielding an area of 0.878 (95% confidence interval: 0.819–0.937) under the receiver operator characteristic (ROC) curve in a field-based analysis (Rauscher et al., 2016). In a patient-based analysis of the same population, the area under the ROC curve for detection of lymph node metastases was 0.732 (95% confidence interval: 0.583–0.850). Some other PSMA-directed agents have not only been linked to positron-emitting isotopes for diagnostic purposes but also to α/β-emitters for radioisotope therapy, e.g., ^177^Lu (Baum et al., 2016). By linking both diagnostic and therapeutic isotopes to the same tracer (i.e., creating ‘theranostic’ agents), one can reliably predict the in-vivo binding of the therapeutic agent and achieve patient- and organ-tailored dose calculation. Recent retrospective European studies have demonstrated promising surrogate response rates for this kind of therapy (Rahbar et al., 2017; Brauer et al., 2017), and future randomized phase II/III trials are warranted to evaluate whether these will ultimately translate into survival benefits. A more detailed description and imaging example of this ‘theranostic’ concept is provided in the paragraph on neuroendocrine tumors later in this article.

^18^F–Fluorodehydrotestosterone (^18^F–FDHT) is a less widely used testosterone analogue binding the androgen receptor, whose activation is essential for the survival and proliferation of prostate cancer cells, and the central target of medical treatment of advanced and recurrent prostate cancer. Due to its specificity for this receptor, ^18^F–FDHT cannot only localize metastases but has also been used for in-vivo visualization of binding of androgen receptor-blocking therapeutic agents (Pandit-Taskar et al., 2016; Vargas et al., 2014). This allows for an early evaluation of drug binding and for adjusting the dose to an individual patient’s needs (Rathkopf et al., 2013). This concept of a patient-tailored ‘biologically relevant dose’ might revolutionize the dosing of anti-cancer drugs, away from the ‘maximum tolerated dose’ concept, which is based on side effects rather than drug efficacy. Another potential application of this tracer is as a predictive biomarker to assess the prospects of pharmacological blockade of the androgen receptor.

### Breast cancer

**Fig. 2:**
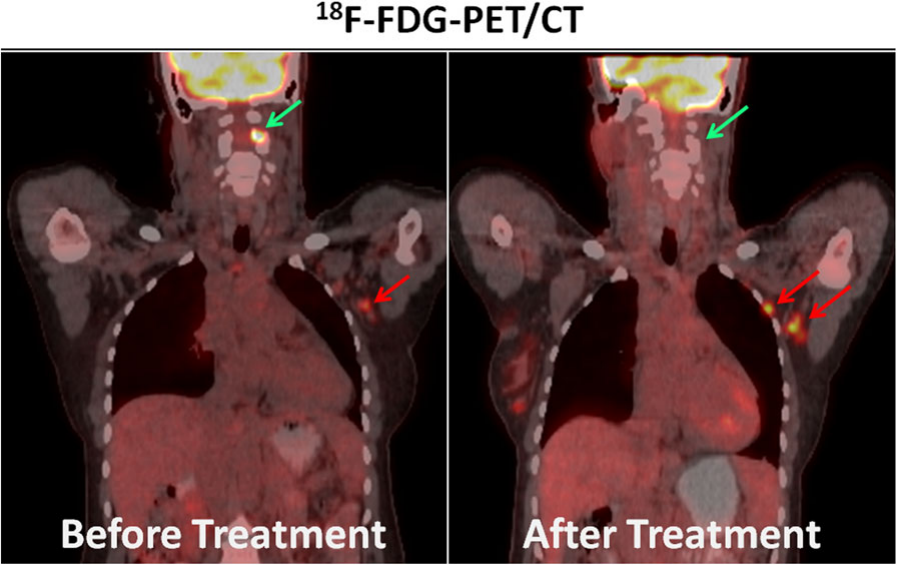
^18^F–FDG-PET/CTs in a patient with metastatic breast cancer before and after systemic therapy illustrating mixed treatment response. While an osseous cervical spine metastasis (green arrows) responded to treatment, several axillary lymph node metastases (red arrows) showed growth and increased FDG uptake. Mixed response to treatment is more commonly encountered in advanced, previously treated malignancies and hybrid imaging may help to prospectively identify patients in need for alternative treatment regimens

**Fig. 3:**
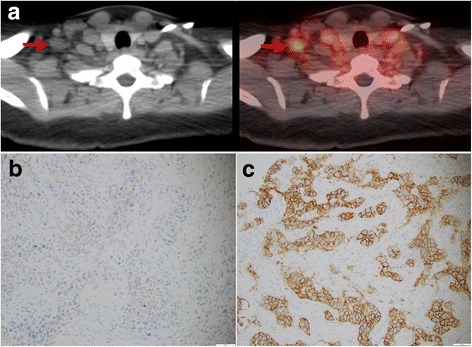
**a** ^89^Zr-Trastuzumab-PET/CT in a patient with HER2-negative primary breast cancer showing intense tracer uptake in a right supraclavicular lymph node metastasis. **b** Immunohistochemistry of the primary cancer proved HER2-negativity, while **c** biopsy and immunohistochemistry of the suspicious lymph node showed markedly HER2-positive metastatic disease. This patient responded to HER2-directed therapy. Adapted and reprinted with permission from Ulaner et al. J Nucl Med 2016;57:1523–1528

Similar to prostate cancer, the FDG avidity of breast cancer is variable and depends on its histologic and biologic characteristics, where invasive ductal carcinomas, higher grade, and triple-negative tumors (i.e. tumors which lack estrogen receptors (ER), progesterone receptors (PR), and human epidermal growth factor receptors 2 (HER2) typically show more intense tracer uptake (Yoon et al., 2014). For diagnostic purposes, it is therefore essential to consider these factors when choosing the appropriate diagnostic tools. For patients with ER-positive, HER2-negative breast cancer, for example, the hybrid application of FDG-PET and CT was shown to reveal distant metastases that were not apparent on conventional imaging or surgical staging in up to 26% of cases (Ulaner et al., 2017). For lobular breast cancer, which is generally less FDG avid than ductal breast cancer, the CT component not only decreases the number of false positive PET findings but also detects non-FDG-avid metastases (Hogan et al., 2015). Due to this heterogenous FDG-avidity of breast cancer, alternative molecular imaging probes targeting receptor molecules have been developed and tested clinically. One of these is radiolabeled Trastuzumab, a monoclonal antibody targeting HER2 (Henry et al., 2017). This drug was approved for clinical use by the FDA in 1998 and is a mainstay of therapy of HER2-positive breast cancer. Of note, in up to 15% of patients with HER2-positive primary cancer, HER2-expression can be lost in metastatic foci (Priedigkeit et al., 2017; Paik et al., 2008). This cancer-evolutionary step renders HER2-directed therapy ineffective and might be one of the reasons for mixed responses to systemic therapies that are often encountered in patients with metastatic breast cancer (an example is shown in Fig. 2). As biopsy of metastases is affected by sampling bias and may miss this crucial intra-individual cancer heterogeneity (Mestel, 2017), HER2-directed hybrid imaging may identify a sub-group of patients in need for alternative treatment. Conversely, about 10–20% of patients with primary HER2-negative breast cancer develop HER2-positive metastases and might benefit from HER2-directed systemic therapy (Priedigkeit et al., 2017; Rossi et al., 2015). Also in this scenario, hybrid imaging can help to identify targets for biology-guided precision biopsy and select the appropriate therapy, as shown in a proof-of concept study where ^89^Zr-trastuzumab-PET/CT demonstrated metastases in 5 of 9 patients with primary HER2-negative metastatic breast cancer (an example is provided in Fig. 3) (Ulaner et al., 2016). Two of these patients had biopsy-proven HER2-positive metastases and responded to HER2-targeted therapy. However, the specificity of trastuzumab PET for HER2 overexpressing breast cancer and the relationship between trastuzumab uptake in-vivo and the immunohistochemistry staining results (which are the accepted standard for treatment with HER-directed therapies) need to be studied further (Ulaner et al., 2016; Ulaner et al., 2017).

**Fig. 4:**
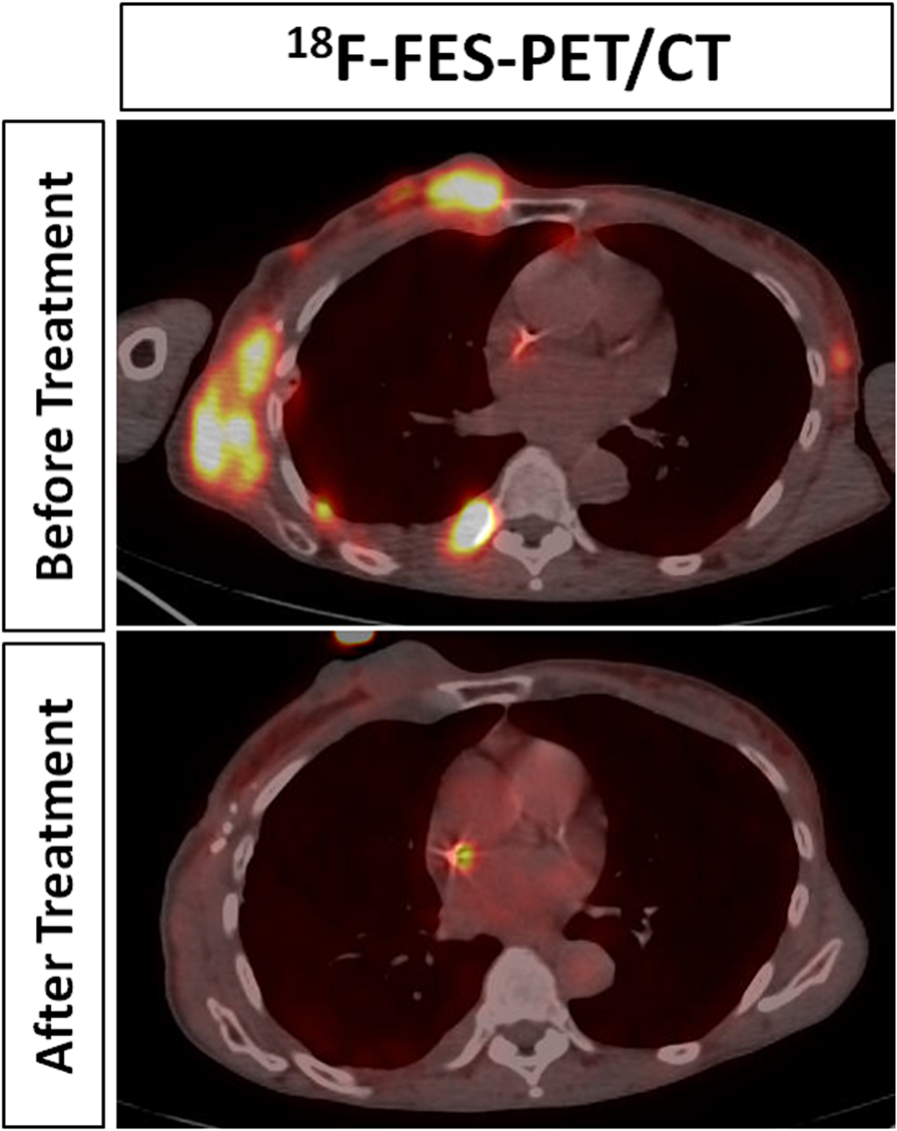
In-vivo monitoring of drug-target-interaction with ^18^F–Fluoro-Estradiol (FES)-PET/CT in a patient with metastatic estrogen receptor-positive breast cancer being treated with an estrogen receptor-directed agent. The pre-therapy scan (top image) showed multiple chest wall metastases with avid tracer uptake. After initiation of the therapy (bottom image), tracer uptake resolved, while the size of the metastases did not change. It is important to recognize that these findings prove that the drug has hit its target but do not necessarily indicate treatment response. Retained tracer is seen in a central venous catheter on both studies

^18^F–Fluoroestradiol (^18^F–FES) is a radiolabeled analogue of estradiol that can be used to image patients with estrogen receptor (ER)-positive breast cancer (Ulaner et al., 2016). As for HER2 expression, significant spatial and temporal inter-metastatic heterogeneity of ER-expression has been reported (Hoefnagel et al., 2013), substantiating the need for repeated whole-body assessment of ER-status by hybrid imaging. In a phase I trial of a novel ER-targeting therapeutic (i.e. GDC0810), ^18^F–FES was also used to monitor in-vivo drug binding and successfully assess the appropriate dose for subsequent phase II trials (Wang et al., 2016). An example of this concept is provided in Fig. 4. This shows that ‘biologically relevant dosing’, as assessed by hybrid imaging, might change the way future clinical oncologic drug trials are designed and conducted.

**Fig. 5:**
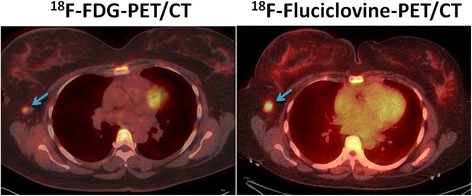
^18^F–Fluorodeoxyglucose (FDG)- and ^18^F–Fluciclovine-PET/CTs in a patient with locally advanced invasive lobular breast cancer. A right axillary lymph node metastasis (blue arrows) showed mild FDG uptake (SUV: 2.1) but more marked Fluciclovine uptake (SUV 5.4)

^18^F–Fluciclovine, which has been approved by the FDA for imaging of patients with recurrent prostate cancer, has also been evaluated in patients with breast cancer. In a prospective clinical trial in patients with locally advanced breast cancer, tracer uptake was seen in 20 of 21 patients with pathologically-proven metastatic axillary lymph nodes and revealed unsuspected extra-axillary metastases in three (Ulaner et al., 2016). Of note, invasive lobular cancer showed higher tracer uptake on ^18^F–Fluciclovine than on ^18^F–FDG-PET/CT (Figure 5), while the converse was true for invasive ductal carcinoma (Ulaner et al., 2016). In a prospective clinical pilot trial of 24 females with newly diagnosed advanced invasive ductal and lobular breast cancer, changes in ^18^F–Fluciclovine uptake before and after neoadjuvant systemic therapy were strongly correlated with treatment response on pathology (Spearman ρ, 0.79, *p* < 0.001) (Ulaner et al., 2017), indicating a potential role in the non-invasive assessment of treatment response for this tracer.

### Neuroendocrine Tumors (NET)

**Fig. 6:**
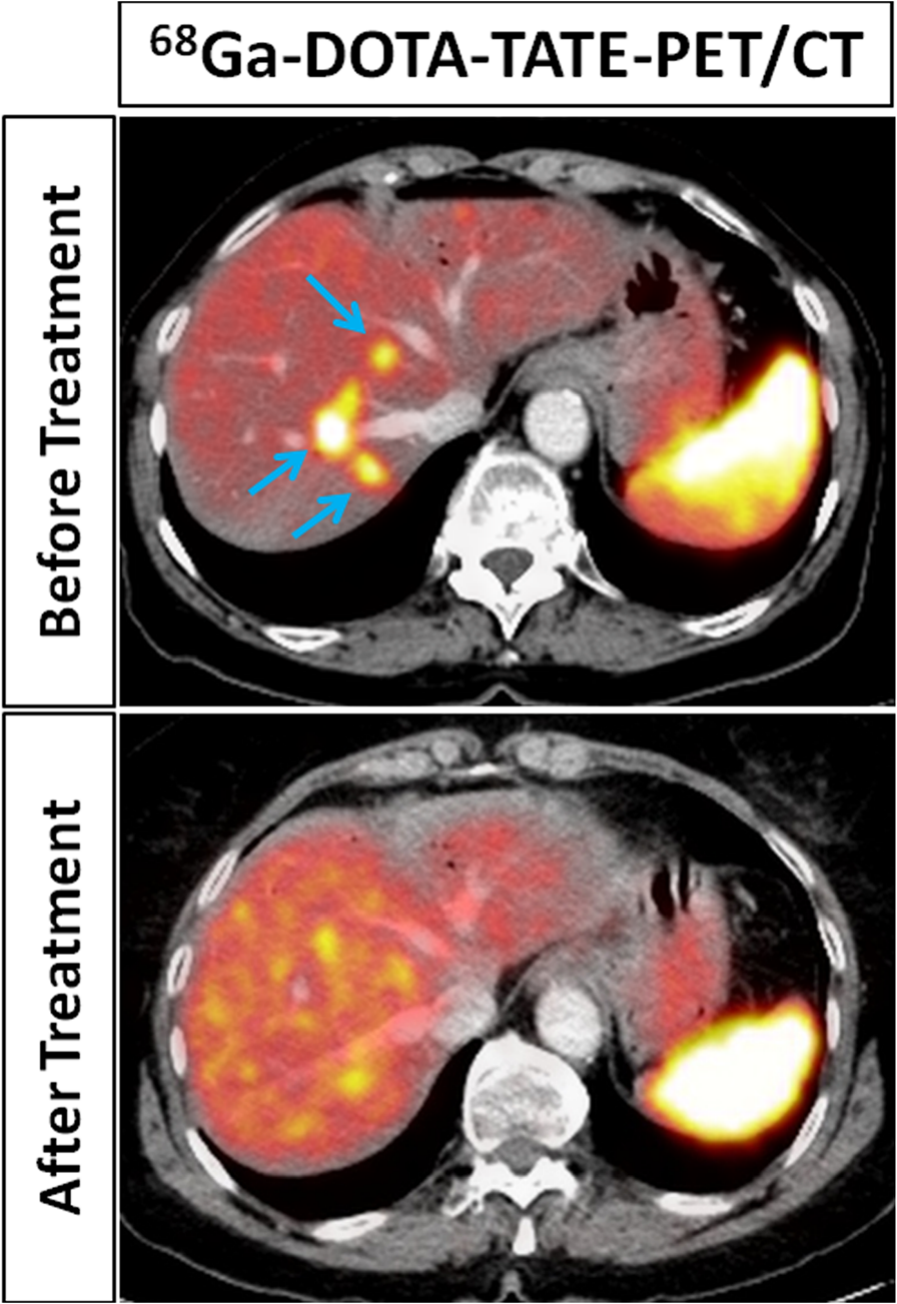
^68^Ga-DOTA-TATE-PET/CTs in a patient with metastatic neuroendocrine tumor before (top row) and after (bottom row) radionuclide therapy with ^177^Lu-DOTA-TATE. The pre-treatment diagnostic scan showed multiple avid liver metastases (arrows), implying that they would also bind the ^177^Lu-labelled therapeutic ‘sister agent’, therefore predicting the treatment response that was ultimately demonstrated on the post-therapeutic PET/CT. Physiologic splenic tracer uptake is seen on both studies

Well-differentiated NETs are typically not FDG avid and overexpress membrane receptors for somatostatin (Baumann et al., 2016). Different types of radiolabeled somatostatin analogues have been developed, including ^111^In-labled agents, and more recently PET-tracers, most of which contain a central ^68^Ga-labeled chelating agent (i.e. DOTA). PET/CT with ^68^Ga-DOTA-TATE was shown to outperform conventional imaging and SPECT/CT with ^111^In-pentetreotide (Sadowski et al., 2016), have a substantial impact on clinical decision making (Calais et al., 2017; Barrio et al., 2017), and was recently approved by the FDA for use in humans with neuroendocrine tumors. The same agent has also been labeled with ^177^Lu, a β-emitter for therapeutic purposes. While the ^68^Ga-labeled compound is being used for localization of cancer foci, it does also predict the uptake and bio-distribution of its ^177^Lu-labeled therapeutic ‘sister’ agent. This has two main implications. First, the diagnostic agent can be used to select patients with avid tumors who will most likely benefit from the radionuclide therapy. Secondly, it allows for the assessment of patient-specific bio-distribution and the calculation of organ doses which determine the maximum tolerable dose (Kairemo & Kangasmaki, 2013). The efficacy of this treatment has recently been demonstrated in a randomized phase III trial in patients with metastatic midgut NET, which showed that the addition of ^177^Lu-DOTA-TATE treatment to best supportive care resulted in a significantly higher response rate (18% vs. 3%, *p* < 0.001) and longer progression-free survival (65.2% vs. 10.8% at 20 months, *p* < 0.001) (Strosberg et al., 2017). An imaging example of this theranostic principle in a patient with metastatic NET is provided in Fig. 6.

## Imaging devices

### PET/CT

**Fig. 7:**
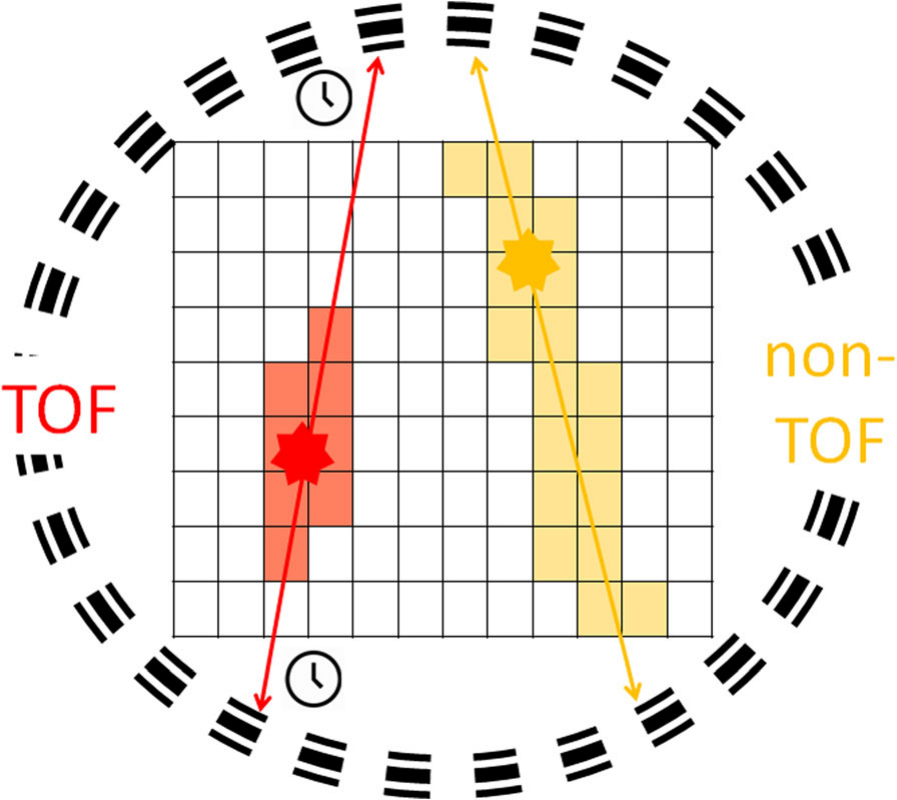
Schematic drawing of the basic principle of time-of-flight (TOF) PET. Annihilation events are marked as stars and the line of response as double-headed arrows. In conventional PET (non-TOF, yellow), the location of the annihilation event cannot be further located on the line of response and can only be deducted by reconstruction. In TOF PET (TOF, red), the time difference between the detection of both γ-rays is recorded, allowing for an estimation of the location of the annihilation event and resulting in a higher signal-to-noise ratio

While the basic technical principle of PET (and PET/CT) has remained unchanged over the years, there are some more recent technical developments which have and probably will become available clinically and have been shown to enhance image quality and speed of data acquisitions. The first is Time-of-Flight (TOF)-PET, which aims to determine the location of annihilation events along the line of response (a schematic drawing of its principles is provided in Fig. 7). The principle of TOF-PET was realized in prototype scanners back in the 1980s (Yamamoto et al., 1982); however, only since the advent of newer detector materials such as lutetium oxyorthosilicate, faster electronics and iterative reconstruction algorithms has the clinical use of TOF-PET been broadly adopted (Vandenberghe et al., 2016). This technique improves the signal-to-noise ratio and thus image quality, especially in heavy patients, low-contrast lesions and areas deep in the body (El Fakhri et al., 2011). Computational strategies to tackle motion-related artifacts are another step towards higher image quality, especially in cases of pulmonary and hepatic lesions, in which tracer uptake is often underestimated due to patient breathing. Various methods for motion correction have been reported (Pepin et al., 2014; Kesner et al., 2016) and it would be beyond the scope of this article to describe all of them in detail. An underlying principle is, however, that motion is captured in the acquired PET data without the need for an external gaiting device (e.g. respiration belts) (Bouyeure-Petit et al., 2017).

### One-minute whole-body PET

Another emerging technology that might result in an unprecedented increase in the sensitivity of PET is ‘one-minute whole-body PET,’ which detects real-time radiotracer uptake at all metastatic tumor sites and organs of the body simultaneously. Current PET systems acquire images in only a 15–20 cm field of view, which renders them relatively insensitive, as more than 90% of the emitted radiation is not used to generate an image. As a result, whole-body PET studies are time consuming and associated with significant radiation exposure, especially when imaging is performed with antibodies that are labeled with long-lived radioisotopes. One-minute whole-body PET, which is being developed under the name “PET Explorer” by Simon Cherry’s PET imaging group at University of California, Davis, has a large axial field of view, which results in a more than 20-fold greater sensitivity than that of current PET/CT devices (Explorer: University of California Davis). Although this greater technical sensitivity does not directly translate into a 20-fold higher clinical sensitivity for pathologic imaging findings, the duration of a whole-body FDG PET scan with the “PET Explorer” could be reduced to 1 min as compared to 20 min with current systems. This improvement will not only make such scans much more convenient for patients but will also markedly reduce motion artifacts, especially those resulting from respiratory motion. As a consequence, the new scanner will make it possible to characterize smaller lesions with much higher reproducibility than is currently possible. The increased sensitivity can be used to acquire scans rapidly, i.e. within a single breath hold to minimize breathing artifacts or, alternatively, to reduce the patient-administered radioactivity 20-fold. The latter is especially compelling in pediatric patients and in studies involving long-lived radiopharmaceuticals (e.g. ^89^Zr), or where it is desired to conduct repeated longitudinal tumor tracking for response assessment. This new generation of PET scanners will also provide a unique capability to conduct a pharmaco-kinetic characterization of any drug able to be tagged with a positron emitter across the entire patient’s disease burden, providing new opportunities for studying and quantifying tumor heterogeneity. The very fast whole-body scans will allow extremely high patient throughput and consolidation of space and personnel. The major challenge for the marketability of this new generation of PET-scanners will be their economic viability in comparison to conventional imaging systems.

### PET/MRI

There are currently two fully integrated commercially available clinical PET/MRI systems on the market and the first integrated PET/MRI was approved by the FDA in 2011. As of 2015, 70 institutions worldwide had at least one PET/MRI system in use, with oncologic imaging being by far the most common application (Fendler et al., 2016). The major advantages of PET/MRI over PET/CT are that it allows for truly simultaneous PET- and MRI-data acquisition and that it saves the patient from the ionizing radiation of the CT component of PET/CT. Compared to separate PET and MRI examinations, the simultaneous procedure could also save time and space and help to streamline a patient’s diagnostic workup. All of that comes at a price; not only is there the initial capital investment, but there are also specific technical challenges, and personnel need to be trained to operate the equipment (Chen & An, 2017). The true added value of simultaneous PET/MRI, as compared to PET/CT and MRI, remains to be determined. We hypothesize that the greatest potential for oncologic PET/MRI lies in organ systems for which MRI is already essential today and where CT offers only limited diagnostic value, e.g. brain tumors, gynecologic malignancies, prostate cancer. For example, Eiber and colleagues from the Technical University of Munich recently demonstrated that simultaneous ^68^Ga-PSMA PET/MRI improved localization of primary prostate cancer within the gland of 53 patients when compared to multiparametric MRI alone (areas under the ROC curves: 0.88 vs. 0.73, *p* < 0.001) (Eiber et al., 2016). Additionally, pediatric patients and cancer survivors on long-term surveillance will benefit from the reduced radiation dose of PET/MRI.

## Education and training

The above described rapidly growing number of approved radiotracers and hybrid imaging modalities necessitates the adaption of training curricula for all types of involved staff. Radiochemists, radiopharmacists, and medical physicists will have to broaden their expertise and constantly adopt new skills as new imaging agents and modalities are approved by regulatory authorities. The training curricula of technologists will probably become more complex, especially with the wider distribution of PET/MRI systems. Also, the training of radiologists and nuclear medicine physicians will become more challenging due to the need to understand the physiology and biochemistry of multiple tracers as well as the complex physics of MRI signals. Another layer of complexity is added with the growing number of theranostic agents and the need for specific training, which is currently almost completely lacking in radiology training curricula. Nuclear medicine physicians will have to dedicate more of their training to MRI, which will be substantially more demanding than mastering CT. Both disciplines will have to intensify their education, training, and understanding of the biochemical and molecular mechanisms underlying these novel imaging techniques. Training a new generation of physicians proficient in both nuclear medicine and diagnostic radiology will be one of the greatest challenges of our time! Furthermore, sub-specialization in organ systems and tumor types will become even more important to provide high-quality and clinically meaningful reports while maintaining high throughput.

## Data analysis

The growing number and complexity of hybrid imaging procedures and the rapidly growing amount of data produced pose several challenges but also open up appealing opportunities for the future. First, these examinations have to be read and reported, increasing the need for properly trained personnel. Second, this vast amount of data is a potentially very rich source of information that remains mostly untapped at this time. At MSKCC, several hundred megabytes of data are generated and stored per patient each year. It has also been estimated that 30% of the entire world’s stored data is in the health care industry (Huesch & Mosher, 2017). A large—and probably the most complex—portion of this data stems from medical imaging. While other data-rich industries, e.g. banking and insurance, invest major resources in big data analysis, health care providers, even academic centers, rarely have employees solely dedicated to data analysis. Such personnel and accompanying infrastructure, however, are needed for advanced analysis techniques, including but not limited to machine learning, artificial intelligence and sophisticated data engineering. The potential opportunities of such analyses are manifold. Algorithms could pre-scan imaging studies to identify anatomical landmarks (Ghayoor & Vaidya, 2017), recognize distinct patterns of normal tissue and common pathologies (Cicero et al., 2017; Summers, 2016), and may allow the physician to focus his or her attention on more complex findings or cases that the computer is not able to classify. In a recently published analysis on chest radiographs, for example, deep convolutional neural networks being trained and validated with studies of 857 patients were found to reliably detect and classify findings of pulmonary tuberculosis in a test population (areas under the ROC-curves of 0.97–0.98) (Lakhani & Sundaram, 2017). Another artificial neuronal network that was trained on 895 women’s mammograms was able to detect breast cancer in 251 test cases with similar accuracy as three radiologists (areas under the ROC-curves: 0.79–0.87 for humans, 0.82 for the network) (Becker et al., 2017). Of note, the radiologists’ readings were consistently less sensitive and more specific than the neural network in this study, indicating that this kind of image analysis could be particularly useful when incorporated into screening algorithms. These algorithms could also be trained to detect urgent imaging findings that require immediate attention, e.g. pneumothorax (Cai et al., 2009) and pulmonary embolism (Bouma et al., 2009), thereby increasing patient safety. Furthermore, feature extraction, machine learning and artificial intelligence/deep learning algorithms could identify findings and patterns that are imperceptible to human cognition, some of which might be clinically useful imaging biomarkers. In patients with mild cognitive impairment, for instance, a MRI-based machine learning algorithm was shown to identify individuals at risk for disease progression to Alzheimer’s disease (area under the ROC-curve of 0.77) (Moradi et al., 2015). Combining this imaging biomarker with clinical information (i.e. baseline cognitive performance, age) further increased the area under the ROC-curve to 0.90. This example demonstrates that imaging data and imaging-derived biomarkers should always be seen and analyzed in the context of a patient’s whole medical record, thereby increasing the chances of finding exploitable correlations.

## Conclusion

Hybrid imaging has revolutionized the diagnosis of a wide range of malignancies at all stages and plays a major role in the modern management of patients with cancer. A growing number of radiotracers and technical advancements in the near future will further widen and strengthen its role as an in-vivo companion diagnostic. It will help to provide individualized patient care that takes into account the inter- and intra-individual, spatial and temporal heterogeneity of cancer, promoting the very realization of precision medicine. Theranostic agents will extend therapeutic options, particularly in patients with metastatic disease. Numerous obstacles remain to be overcome, most importantly that of providing the human resources and skills needed to excel in such a rapidly evolving and expanding field. The management and meaningful use of big imaging data and its linkage to other information sources is another challenge but also a huge opportunity for producing future discoveries that should lead to improved patient care and outcomes.

## Abbreviations

CT: Computed tomography-CT
ER: Estrogen receptor
FDA: Food and drug Administration
FDG: ^18^F–fluorodeoxyglucose
FDHT: ^18^F–Fluorodehydrotestosterone
FES: ^18^F–Fluoroestradiol
HER2: Human epidermal growth factor receptor 2
MRI: Magnetic resonance imaging
NaF: ^18^F–sodium fluoride
NET: Neuroendocrine tumor
PET: Positron emission tomography
PSA: Prostate-specific antigen
PSMA: Prostate-specific membrane antigen
ROC: Receiver operator characteristic
SPECT: Single photon emission computed tomography
TOF: Time--of-flight
